# AlignStat: a web-tool and R package for statistical comparison of alternative multiple sequence alignments

**DOI:** 10.1186/s12859-016-1300-6

**Published:** 2016-10-26

**Authors:** Thomas Shafee, Ira Cooke

**Affiliations:** 1 Department of Biochemistry and Genetics, La Trobe Institute for Molecular Science, La Trobe University, Melbourne, 3086 Australia; 2 Department of Molecular and Cell Biology, James Cook University, Townsville, 4811 Australia

## Abstract

**Background:**

Alternative sequence alignment algorithms yield different results. It is therefore useful to quantify the similarities and differences between alternative alignments of the same sequences. These measurements can identify regions of consensus that are likely to be most informative in downstream analysis. They can also highlight systematic differences between alignments that relate to differences in the alignment algorithms themselves.

**Results:**

Here we present a simple method for aligning two alternative multiple sequence alignments to one another and assessing their similarity. Differences are categorised into merges, splits or shifts in one alignment relative to the other. A set of graphical visualisations allow for intuitive interpretation of the data.

**Conclusions:**

AlignStat enables the easy one-off online use of MSA similarity comparisons or into R pipelines. The web-tool is available at AlignStat.Science.LaTrobe.edu.au. The R package, readme and example data are available on CRAN and GitHub.com/TS404/AlignStat.

**Electronic supplementary material:**

The online version of this article (doi:10.1186/s12859-016-1300-6) contains supplementary material, which is available to authorized users.

## Background

Multiple sequence alignments (MSAs) aim to organise a set of sequences by placing homologous residues into columns, and their accuracy affects subsequent steps in bioinformatic pipelines such as phylogenetic inference [1] and protein structure prediction [2]. However, since there is no objective function to measure true ‘biological correctness’ of an alignment, an array of alternative methods exist based on different assumpitions. These algorithms often make different alignment predictions [2], especially in MSAs with many insertions and deletions, for example in cysteine-rich proteins. Quantitative comparison and intuitive visualisation of alternative MSAs can help users make decisions as to which regions are generally agreed upon and whether any regions should be removed in further analyses. Quantitative similarity measures are also used when assessing the accuracy of alignment algorithms against benchmark MSAs, either synthetically generated [3, 4] or a curated database [5, 6], and to refine phylogenies [7].

A common method of alignment comparison is though a combination of the sum of pairs score (SPS), and total column score (CS) [8]. The sum of pairs score measures what proportion of all residue pairs within columns of one alignment are retained in a comparison alignment and the total column score measures the proportion of columns where both alignments agree completely (ie for all sequences). These methods have the benefit of including all homology information in a single score, however their interpretation can be hampered by the fact that they scale non-linearly with the degree of similarity at a site (Additional file 1: Figure S1).

Here, we use a complementary method based on a matrix of equivalency functions to allow comparable quantification of both similarity and of alternative sources of dissimilarity. Each position in the matrix corresponds to a residue of the reference MSA, with an equivalency function indicating its relationship to the corresponding residue in the comparison MSA. We present a simple set of quantitative measures and graphical visualisations for interpreting MSA comparisons. An R package generates a standardised set of comparison matrices and scores for analysis pipelines and graphing, and a user-friendly web-tool interface enables easy one-off use.

## Implementation

### Quantifying similarity

When alignment algorithms make different homology predictions for a set of sequences, the columns of the resulting MSAs will contain different residues. The AlignStat R package contains functions for calculating all MSA comparison statistics and creating plots quantifying differences in a manner that is equivalent for nucleotide or amino acid sequences.

Each MSA of *n* sequences is treated as a matrix of characters (residues plus a gap character) with the same number of rows. The two matrices are therefore defined as *P* (of dimensions *n* x *p*) and *Q* (of dimensions *n* x *q*), where each row represents an aligned sequence. Residues can occur multiple times in a sequence and so are numbered by occurrence such that each character in a row has a unique designation (Additional file 1: Figure S4). This ensures that alignment columns that contain a non-homologous occurrence of a residue are correctly distinguished. For the matrices *P* and *Q*, each column vector pair ***p***_***i***_, ***q***_***j***_ is compared to calculate the similarity measure *S*_*ij*_ defined in Eq. 1 (where ***p***_***i***_ is the *i*th column of *P*, and ***q***_***j***_ is the *j*th column of *Q*).

1 $$ {S}_{ij} = \frac{1}{n}{\displaystyle \sum_{x=1}^n}\ \varepsilon \left({P}_{xi},{Q}_{xj}\right) $$

Where *S*_*ij*_ is the similarity score for each column pair between *P* and *Q*, the equivalency function ε is defined in Eq. 2.

2 $$ \varepsilon \left(a,b\right)\left\{\begin{array}{c}\hfill 1\kern0.5em  if\kern0.5em a\kern0.5em =\kern0.5em b\kern0.5em \wedge \kern0.5em a\kern0.5em \ne \kern0.5em "-"\hfill \\ {}\hfill 0\kern0.5em  otherwise\hfill \end{array}\right. $$

The similarity matrix ***S*** can be visualised using the *plot_similarity_heatmap* function of the AlignStat R package. Evaluating ***S*** is the most computationally expensive calculation in the AlignStat scoring method and has been implemented in C++ for maximum efficiency.

#### Detailed match scoring for comparable MSA columns

For each column in *P* we find its “match” in *Q* by finding the index j at which *S*_*ij*_ is maximized. The match between columns, *P*_*i*_ and *Q*_*j*_ is then categorised leading to the dissimilarity matrix, *D* (of dimensions *n* x *p* x *5*) based on the functions defined in Eq. 3 and Eq. 4. This matrix categorises five types of outcome when the reference and comparison alignments are compared. It is called the dissimilarity matrix because four of the five alternatives correspond to various types of mismatch.

3 $$ {D}_{xik} = {\varepsilon}_k\left({P}_{xi},{Q}_{xj}\right) $$

Where *ε*_*k*_ *(a,b)* is the kth equivalency function as defined in Eq. 4.

4 $$ \begin{array}{l}{\varepsilon}_1\left(a,b\right)\kern0.5em \left\{\begin{array}{c}\hfill 1\kern0.5em  if\ a=b\kern0.5em \wedge \kern0.75em a\kern0.5em \ne "-"\hfill \\ {}\hfill 0\  otherwise\hfill \end{array}\right.\\ {}{\varepsilon}_2\left(a,b\right)\kern0.5em \left\{\begin{array}{c}\hfill 1\kern0.5em  if\ a=b\kern0.5em \wedge \kern0.5em a="-"\hfill \\ {}\hfill 0\  otherwise\hfill \end{array}\right.\\ {}{\varepsilon}_3\left(a,b\right)\kern0.5em \left\{\begin{array}{c}\hfill 1\  if\ a\ne b \wedge b="-"\hfill \\ {}\hfill 0\  otherwise\hfill \end{array}\right.\\ {}{\varepsilon}_4\left(a,b\right)\kern0.5em \left\{\begin{array}{c}\hfill 1\kern0.5em  if\ a\ne b\kern0.5em \wedge \kern0.5em a="-"\hfill \\ {}\hfill 0\  otherwise\hfill \end{array}\right.\\ {}{\varepsilon}_5\left(a,b\right)\kern0.5em \left\{\begin{array}{c}\hfill 1\kern0.5em  if\ a\ne b\kern0.5em \wedge \kern0.5em a\kern0.5em \ne "-" \wedge \kern0.5em b\kern0.5em \ne "-"\hfill \\ {}\hfill 0\  otherwise\hfill \end{array}\right.\end{array} $$

Where the five *ε*_*k*_ *(a,b)* are equivalency functions (see supplementary information for formal definitions) with the following meanings. The first equivalency (*ε*_*1*_) is a ‘match’, in which the two characters are identical *and* not gaps. The second equivalency (*ε*_*2*_) is a ‘conserved gap’, when the both characters are gaps. A ‘merge’ is when *P* contains a gap, but *Q* contains any other character (*ε*_*3*_). Similarly, a ‘split’ is when *Q* contains a gap, but *P* contains any other character (*ε*_*4*_). Finally, a ‘shift’ is when two characters are not identical *and* neither are gaps (*ε*_*5*_). The *D* matrix is visualised using the *plot_dissimilarity_matrix* function of the *AlignStat* R package.

#### Summary statistics

The column averages of *D* are used to describe the sources of dissimilarity between the reference and comparison alignments at each alignment position and each equivalency, k. This leads to the results matrix *R* (of dimensions *5* x *p*) defined by Eq. 5.

5 $$ {R}_{ki}=\frac{1}{n}{\displaystyle \sum_{x=1}^n}{D}_{xi\mathrm{k}} $$

Where *R* is the results matrix, each row of which is used to summarise a source of dissimilarity from the *D* matrix.

The match row of the *R* matrix (*R*_1*i*_) is visualised using the *plot_similarity_summary* function of the *AlignStat* R package. The merge, split and shift rows of the *R* matrix (*R*_3*i*_, *R*_4*i*_ and *R*_5*i*_) are referred to collectively as dissimilarities in AlignStat. They are visualised using the *plot_dissimilarity_summary* function.

A single, overall similarity score describes the weighted average similarity of the two MSAs, as defined in Eq. 6. The treatment of gaps in MSAs is complex [9, 10]. In this case, the most instructive measure is to exclude conserved gaps, to prevent results being skewed by the “similarity” of conserved gaps in low occupancy columns. Therefore, the overall score is the sum of the match characters as a proportion of characters that are not conserved gaps. A more stringent column score can also be calculated as the proportion of all columns that have a perfectly identical between the MSAs. A full worked example of the mathematical implementation is available in Additional file 1.

6 $$ score=\frac{\frac{1}{p}{\displaystyle {\sum}_{i=1}^p}{R}_{1i}}{1-\frac{1}{p}{\displaystyle {\sum}_{i=1}^p}{R}_{2i}} $$

Released versions of the R package are available through the comprehensive R archive network (CRAN) and active development versions are available on github (GitHub.com/TS404/AlignStat). In order to allow AlignStat to scale to large MSAs and provide an acceptable run time the core calculation of equivalency functions and scoring statistics was implemented in C++ using the Rcpp framework [11]. A simple web interface to the AlignStat R package is implemented by the Shiny framework and is available at AlignStat.Science.LaTrobe.edu.au. The source code for the user interface is available at Github.com/iracooke/AlignStatShiny.

## Results and discussion

### R package and example

**Fig. 1:**
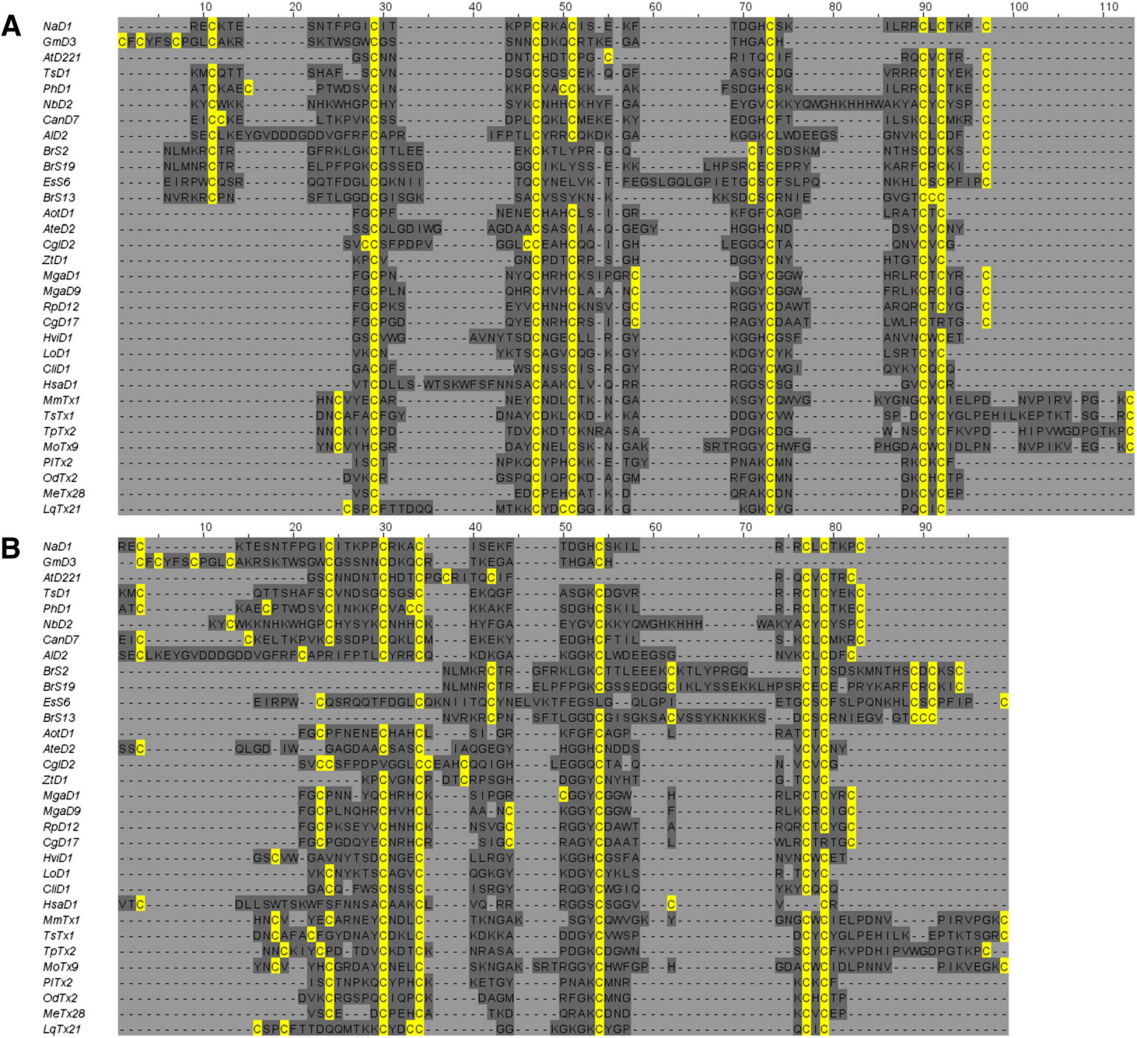
Example alignments of 32 defensin sequences coloured with cysteines in *yellow*, gaps in *light grey*, and all other residues in *dark grey*. **a** Reference alignment generated by CysBar method. **b** comparison alignment generated by ClustalΩ

The *AlignStat* R package contains a *compare_alignments* function to calculate the similarity and dissimilarity matrices, and a set of plotting functions to graphically visualise the results. The main *compare_alignments* function reads input alignments (fasta, clustal, msf, or phylip formats) and outputs a Pairwise Alignment Comparison (PAC) object that contains the matrices and summary information. The example here is a reference MSA of cis-defensin sequences (short, divergent, cysteine-rich proteins [12]) aligned with the CysBar method, which is optimised for highly divergent cysteine-rich proteins [13], compared to an alignment by ClustalΩ [14] (Fig. 1).

**Fig. 2:**
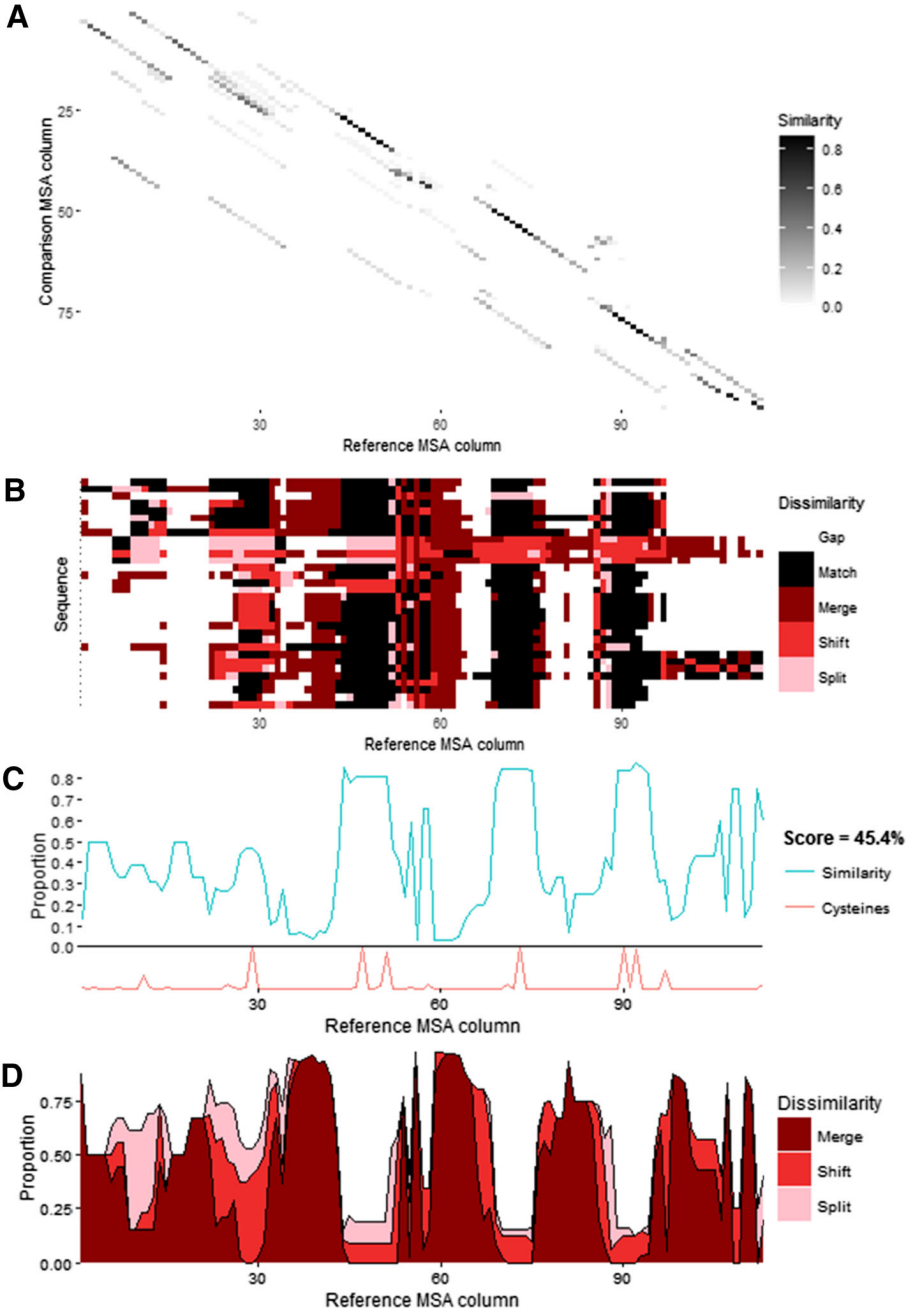
Plots of the similarity (*S*), difference (*D*) and results (*R*) matrices generated by compare_alignments of defensin protein MSAs (reference = CysBar alignment, comparison = ClustalΩ alignment). **a** Similarity matrix visualised by the plot_similarity_heatmap function. **b** Dissimilarity matrix visualised by the plot_dissimilarity_matrix function. **c** Matches in results matrix visualised by the plot_similarity_summary function. **d** Merges, splits and shifts in results matrix visualised by the plot_dissimilarity_proportions function

The *plot_similarity_heatmap* function generates a heatmap of the similarity matrix *S* (Fig. 2a), analogous to a dot-plot graph used to summarise pairwise sequence alignments [15]. Similarity between each column of the two MSAs is shown such that dark diagonal lines indicate regions of high consensus, with regions of potential conflict as parallel grey lines.

A discrete character heatmap of the dissimilarity matrix *D* is generated by the *plot_dissimilarity_matrix* function (Fig. 2b). The reference MSA is arranged on the x-axis with sequences arranged on the y-axis. For each character of the reference alignment, the heatmap colour reports whether it is a match, merge, split, shift, or conserved gap. This indicates how sequence regions (columns) or sequence sets (rows) differ between the MSAs.

The similarity of the MSAs is summarised as a line graph by the *plot_similarity_summary* function (Fig. 2c). The average column match is shown for each reference MSA column, normalised to the proportion of characters that are not gaps. Cysteine proportion can also optionally be reported, since the alignment accuracy of cysteine-rich proteins often correlates with key cysteine motifs. Likewise, a stacked area plot summarising the sources of dissimilarity is generated by the *plot_dissimilarity_summary* function (Fig. 2d). It presents the average merge, split and shift occurrence for each reference MSA column, also normalised to proportion of characters that are not gaps.

When a ‘true’ reference alignment is known (either simulated, or manually curated) the overall similarity statistics can be used to compare which alternative alignment methods most accurately recreate the reference MSA, and the columnwise similarity statistics indicate the causes of any discrepancies. In this case, higher scores indicate a higher recapitulation by the comparison alignment of the homologous residues in the reference alignment. When the ‘true’ alignent is unknown, as is often the case for real datasets, then the similarity statistics quantify consensus and uncertainty between the alignments. In this case, columns with higher scores indicate agreement of which residues are agreed upon as homologous between the two MSAs. Low scores indicate significant discrepancies, which may occur due to repeat regions, insertions and deletions, or low conservation.

For the defensin example, the highest similarity between the MSAs clusters around the conserved cysteine columns. However, misalignment of non-homologous cysteines and frequent merger of low occupancy inter-cysteine regions by ClustalΩ lead to a similarity score of 45.5 %. The splitting of cysteine columns in the defensin alignment by ClustalΩ indicates which loop insertions and deletions prevent the algorithm from finding true structurally homologous cysteines. In this case, cysteines were split from one column to be merged in with non-homologous cysteines. Similarly, cysteines at the N-terminal end of the proteins are are erroneously split, losing information on their homology. Additionally, an entire set of four sequences was clearly translocated to the right, misaligning all cysteines and inter-cysteine regions. These differences in predicted homology significantly affect any phylogenetics or structure homology modelling using the alignment. By comparison, a ClustalΩ alignment of conserved S1 proteases differs only by minor translocations (similarity score of 81 %) compared to the curated benchmark BALI alignment [5] (Additional file 1: Figures S2 and S3). This reflects far higher reproduction of the curated S1 protease reference alignment by ClustalΩ, particularly in the strucurally conserved protein core regions.

### Online web-tool

A webserver at AlignStat.Science.LaTrobe.edu.au performs the *AlignStat* method and outputs the set of graphs generated by the R script *plot* functions described above. The matrices and output graphs can then be downloaded. Example data is also provided to perform a test run. The server is capable of performing the method on MSAs of up to 1000 sequences, each with 1000 alignment columns. Additionally, both online and offline versions of AlignStat can compute and visualise sum of pairs analyses of alignments (Additional file 1: Figure S3). This online web-tool implementation allows for easy use of the method without needing to be familiar with the R programming.

## Conclusions

The online and offline *AlignStat* tools allow the quantitative comparison and graphical interpretation of alternative MSAs of a set of sequences. Summarising similarity and dissimilarity aids interpretation of alternative MSAs. In particular understanding the differences between two MSAs can demonstrate significantly different homology predictions for important residues. These measures therefore complement and extend existing offline sum of pairs tools such as SuiteMSA and MQAT [16, 17]. The R package function can be placed into analysis pipelines, and the online web-tool provides a user-friendly graphical interface.

## Availability and requirements

**Project name:** AlignStat

**Project home page:** AlignStat.Science.LaTrobe.edu.au

**Repository:** GitHub.com/TS404/AlignStat

**Operating system(s):** Platform independent

**Programming language:** R

**Other requirements:** R 3.1 or higher

**License:** Academic Free License 3.0

## Additional file

Additional file 1: Supplementary methods and figures. Worked example of full mathematical implementation for a 6x4 MSA. Supplementary **Figures S1**–**S8.** on comparison of scores and additional S1 protease family example. (PDF 1300 kb)

## Abbreviations

MSA: Multiple sequence alignment
PAC: Pairwise Alignment Comparison
SPS: Sum of pairs score
CS: Total column score
CRAN: Comprehensive R archive network
