# Supplementary material for: AlignStat: a web-tool and R package for statistical comparison of alternative multiple sequence alignments
Source: BMC Bioinformatics. 2016 Oct 26;17:434. doi: 10.1186/s12859-016-1300-6 (PMC5081975; doi:10.1186/s12859-016-1300-6)
Supplement: Additional file 1: — Supplementary methods and figures. Worked example of full mathematical implementation for a 6x4 MSA. Supplementary Figures S1–S8. on comparison of scores and additional S1 protease family example. (PDF 1300 kb) [file 12859_2016_1300_MOESM1_ESM.pdf]

Supplementary material

AlignStat: A web-tool and R package for statistical comparison of alternative multiple sequence alignments

Thomas M A Shafee[1]* and Ira R Cooke[2,1]

[1] Department of Biochemistry, La Trobe Institute of Molecular Sciences, La Trobe University, Melbourne 3086, Australia

[2] Department of Molecular and Cell Biology, James Cook University, Townsville 4811, Australia

*Correspondence: T.Shafee@latrobe.edu.au

Contents

Mathematical Implementation 2

  Quantifying similarity and identifying the most comparable MSA columns 2

  Quantifying dissimilarity 2

Supplementary Figures 5

  Figure S1 | Effect of triangular numbers on column scores 5

  Figure S2 | Output graphs for S1 family protease protein MSAs 7

  Figure S3 | Comparison of output graphs for defensin and S1 family protease protein MSAs 8

  Figure S4 | Generating a numbered-character sequence alignment 9

  Figure S5 | Generating a similarity matrix 10

  Figure S6 | Generating a dissimilarity matrix 11

  Figure S7 | Generating a results matrix 12

  Figure S8 | Generating an overall similarity score 13

Mathematical Implementation

Quantifying similarity and identifying the most comparable MSA columns

When alignment algorithms make different homology predictions for a set of sequences, the columns of the resulting MSAs will contain different residues. The method for quantitative, pairwise comparison between one MSA with respect to a reference MSA is equivalent for nucleotide or amino acid sequences.

Each MSA of *n* sequences is treated as a matrix of characters (residues plus a gap character) with the same number of rows. The two matrices are therefore defined as P (of dimensions *n* x *p*) and Q (of dimensions *n* x *q*), where each row represents an aligned sequence. Residues can occur multiple times in a sequence and so are numbered by occurrence such that each character in a row has a designation (**Figure S4**). This ensures that alignment columns that contain a non-homologous occurrence of a residue are correctly distinguished.

The first step in comparing the two alternative MSAs is to identify the most comparable columns. For the matrices P and Q, each column vector pair $\boldsymbol{p}_i, \boldsymbol{q}_j$ is compared to calculate the similarity measure $\boldsymbol{p}_i, \boldsymbol{q}_j$ defined in **Equation 1** (where $\boldsymbol{p}_i$ is the ith column of P, and $\boldsymbol{q}_j$ is the jth column of Q). The similarity matrix, S (of dimensions *p* x *q*), measures the similarity between the columns of P and Q (**Figure S5**). The S matrix is visualised using the *plot_similarity_heatmap* function of the AlignStat R package.

$$S_{ij} = \frac{1}{\text{n}} \sum_{x=1}^{n} \varepsilon(P_{xi}, Q_{xj})$$

Equation 1
Where S_{ij} is the similarity score for each column pair between *P* and *Q*, the equivalency function ε is defined in **Equation 2**.

$$\varepsilon(a,b) \begin{cases} 1\ if\ a = b\ \wedge\ a \neq "-" \\ 0\ otherwise \end{cases}$$

Equation 2

Quantifying dissimilarity

For each column in P we find its "match" in Q by finding the index j at which S_{ij} is maximized. The match column in Q is analysed in more detail to categorise the causes of difference. This leads to the dissimilarity matrix, D (of dimensions *n* x *p* x *5*) based on the function defined in **Equation 3**. This dissimilarity matrix categorises the types of difference between the reference and comparison alignments.

$$D_{xik} = \varepsilon_k(P_{xi}, Q_{xj})$$

Equation 3

Where $\varepsilon_k(a,b)$ is the kth equivalency function as defined in **Equation 4**.

$$\varepsilon_1(a,b) \begin{cases} 1\ if\ a = b\ \wedge\ a \neq "-" \\ 0\ otherwise \end{cases}$$

$$\varepsilon_2(a,b) \begin{cases} 1\ if\ a = b\ \wedge\ a = "-" \\ 0\ otherwise \end{cases}$$

$$\varepsilon_3(a,b) \begin{cases} 1\ if\ a \neq b\ \wedge\ b = "-" \\ 0\ otherwise \end{cases}$$

$$\varepsilon_4(a,b) \begin{cases} 1\ if\ a \neq b\ \wedge\ a = "-" \\ 0\ otherwise \end{cases}$$

$$\varepsilon_5(a,b) \begin{cases} 1\ if\ a \neq b\ \wedge\ a \neq "-"\ \wedge\ b \neq "-" \\ 0\ otherwise \end{cases}$$

Equation 4

The first equivalency (ε_1) is a 'match', in which the two characters are identical *and* not gaps. The second equivalency (ε_2) is a 'conserved gap', when the both characters are gaps. A 'merge' is when *P* contains a gap, but *Q* contains any other character (ε_3). Similarly, a 'split' is when *Q* contains a gap, but *P* contains any other character (ε_4). Finally, a 'shift' is when two characters are not identical *and* neither are gaps (ε_5) (**Figure S6**). The D matrix is visualised using the *plot_dissimilarity_matrix* function of the *AlignStat* R package.

Summarising similarity and dissimilarity

For each k of the D matrix, the column averages are used to describe the sources of dissimilarity between the reference and comparison alignments. The results matrix R (of dimensions *5* x *p*) therefore measures the average dissimilarities between columns of P and Q as defined by the function in **Equation 5** (**Figure S7**).

$$R_{ki} = \frac{1}{n}\sum_{x=1}^{n} D_{xik}$$

Equation 5

Where R is the results matrix, each row of which is used to summarise a source of dissimilarity from the D matrix.

A single, overall similarity score describes the weighted average similarity of the two MSAs, as defined in **Equation 6**. The treatment of gaps in MSAs is complex (Simmons and Ochoterena 2000; Egan and Crandall 2008). In this case, the most instructive measure is to exclude conserved gaps, to prevent results being skewed by the "similarity" of conserved gaps in low occupancy columns. Therefore, the overall score is the sum of the match characters as a proportion of characters that are not merely conserved gaps (**Figure S8**). The match row of the R matrix (R_{1i}) is visualised using the *plot_similarity_summary* function of the *AlignStat* R package. The merge, split and shift rows of the R

matrix (R_{3i}, R_{4i} and R_{5i}) are visualised using the *plot_dissimilarity_summary* function of the *AlignStat* R package.

$$score = \frac{\frac{1}{p}\sum_{i=1}^{p} R_{1i}}{1 - \frac{1}{p}\sum_{i=1}^{p} R_{2i}}$$

Equation 6

Overall similarity score

Supplementary Figures

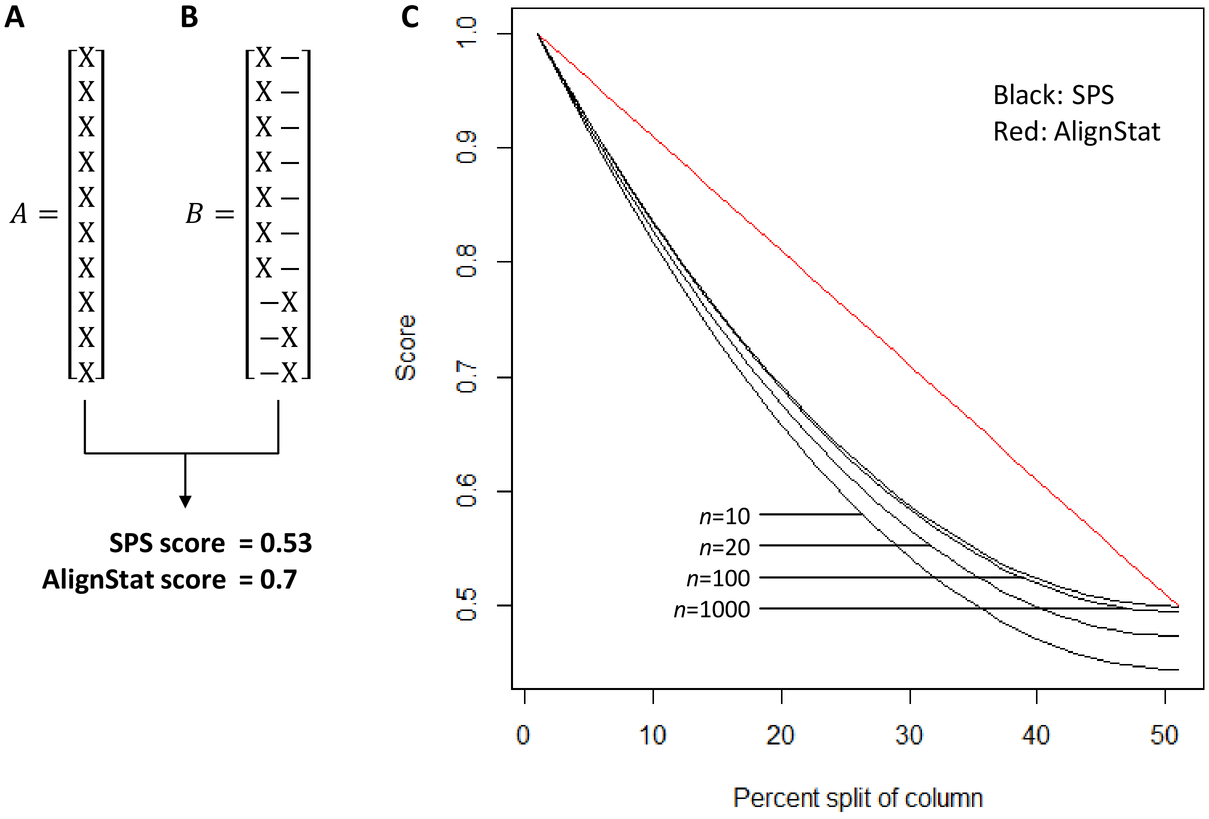

Figure S1 | Effect of non-linearity on sum of pairs scores

(**A**) is an example column of *n*=10 rows. (**B**) is an example where three of the ten residues are split into a separate column. The AlignStat score comparing *A* and *B* is 0.7, reflecting the similarity between the column of alignment *A* and the first column of alignment *B*. The SPS score comparing *A* and *B* is $0.53 = \frac{(3\times2)+(7\times6)}{(10\times9)}$, reflecting the non-linear loss of pairwise connections between residues in the column of alignment *A* when three of them are split into a separate column in alignment *B*. (**C**) This difference in the two measures can be seen as a non-linear relationship between a column split and the SPS score, whereas the AlignStat score has a linear relationship. The SPS score also changes depending on the number of sequences in the alignment (*n*), especially notable when *n*<100.

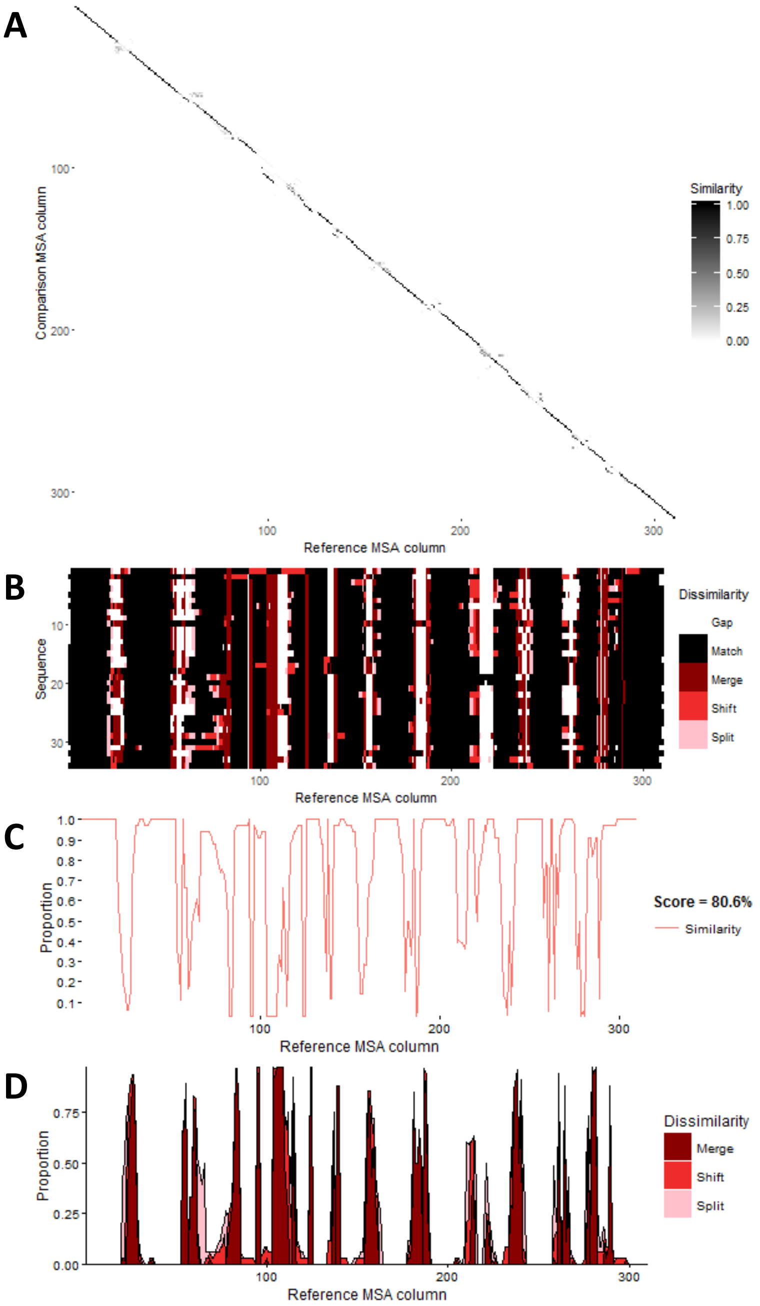

Figure S2 | Output graphs for S1 family protease protein MSAs

Visualisation of the similarities and differences between two alternative MSAs of a set of protease protein sequences (reference = BALI12034 benchmark alignment, comparison = ClustalΩ). Note that similarity is much higher than for the defensin MSA example. (**A**) Similarity matrix visualisation by the *plot_similarity_heatmap* function. (**B**) Dissimilarity matrix visualisation by the *plot_dissimilarity_matrix* function. (**C**) Results matrix (Match row) visualisation by the *plot_similarity_summary* function. (**D**) Results matrix (Merge, Split and Shift rows) visualisation by the *plot_dissimilarity_proportions* function.

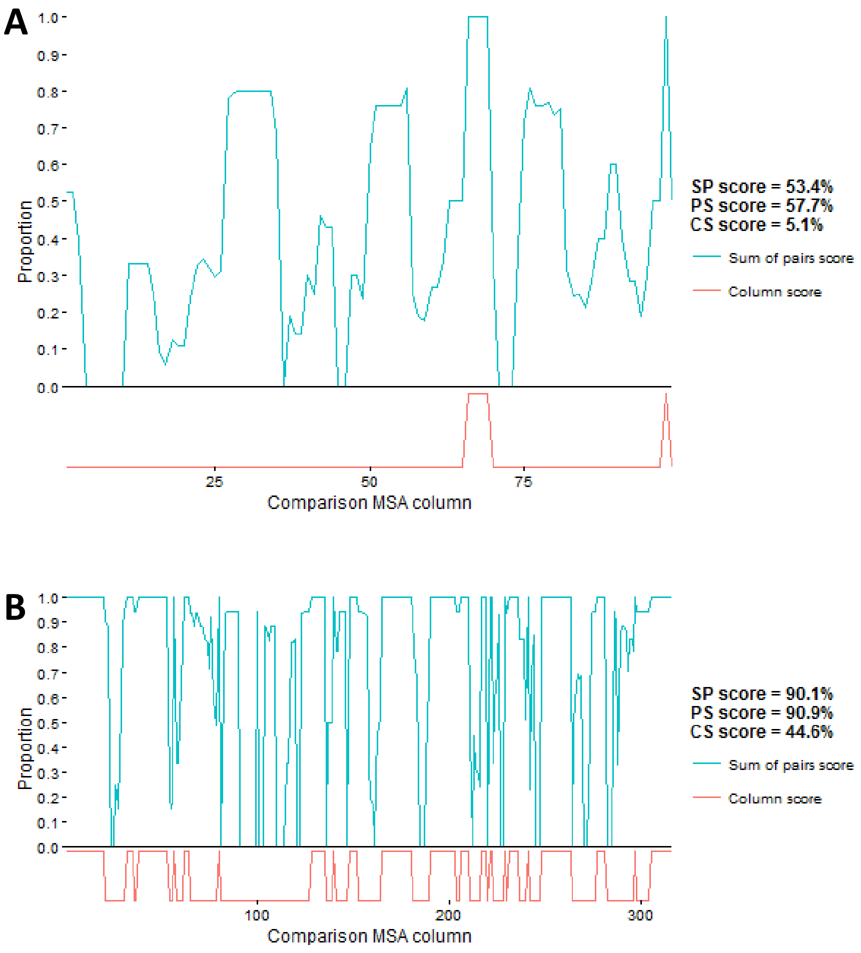

Figure S3 | Comparison of sum-of-pairs graphs for defensin and S1 family protease protein MSAs

Visualisation of the columnwise sum of pairs scores and column scores by the *plot_SP_summary* for (**A**) defensin protein MSAs (reference = CysBar alignment, comparison = ClustalΩ alignment) and (**B**) S1 family protease protein MSAs (reference = BALI12034 benchmark alignment, comparison = ClustalΩ).

Alignment **Numbered alignment**

$$A = \begin{bmatrix} -\text{BCBCD} \\ -\text{CCBCD} \\ \text{ABCB}-- \end{bmatrix} \longrightarrow P = \begin{bmatrix} - & \text{B1} & \text{C1} & \text{B2} & \text{C2} & \text{D1} \\ - & \text{C1} & \text{C2} & \text{B1} & \text{C3} & \text{D1} \\ \text{A1} & \text{B1} & \text{C1} & \text{B2} & - & - \end{bmatrix}$$

Figure S4 | Generating a numbered-character sequence alignment

A sequence alignment (*A*), where each row represents an aligned sequence, is converted to a matrix of numbered characters (*P*), where each character is numbered based on the occurrence of that character occurrence in that row.

First alignment

$$P = \begin{bmatrix} - & \mathrm{B1} & \mathrm{C1} & \mathrm{B2} & \mathrm{C2} & \mathrm{D1} \\ - & \mathrm{C1} & \mathrm{C2} & \mathrm{B1} & \mathrm{C3} & \mathrm{D1} \\ \mathrm{A1} & \mathrm{B1} & \mathrm{C1} & \mathrm{B2} & - & - \end{bmatrix}$$

Second alignment

$$Q = \begin{bmatrix} - & \mathrm{B1} & \mathrm{C1} & \mathrm{B2} & \mathrm{C2} & \mathrm{D1} \\ - & \mathrm{C1} & \mathrm{C2} & \mathrm{B1} & \mathrm{C3} & \mathrm{D1} \\ \mathrm{A1} & - & - & \mathrm{B1} & \mathrm{C1} & \mathrm{B2} \end{bmatrix}$$

$$\longrightarrow S_{ij} = \frac{1}{n}\sum_{x=1}^{n} \varepsilon(P_{xi}, Q_{xj}) \longrightarrow$$

Column similarity

$$S = \begin{bmatrix} 0.33 & 0 & 0 & 0 & 0 & 0 \\ 0 & 0.67 & 0 & 0 & 0 & 0 \\ 0 & 0 & 0.67 & 0 & 0 & 0 \\ 0 & 0.33 & 0 & 0.67 & 0 & 0 \\ 0 & 0 & 0.33 & 0 & 0.67 & 0 \\ 0 & 0 & 0 & 0.33 & 0 & 0.67 \end{bmatrix}$$

Figure S5 | Generating a similarity matrix

The columns of two matrices (*P* and *Q*) are compared using the similarity function defined in **Equation 1** to generate the similarity matrix (*S*), which describes the proportion of identical characters in each column pair.

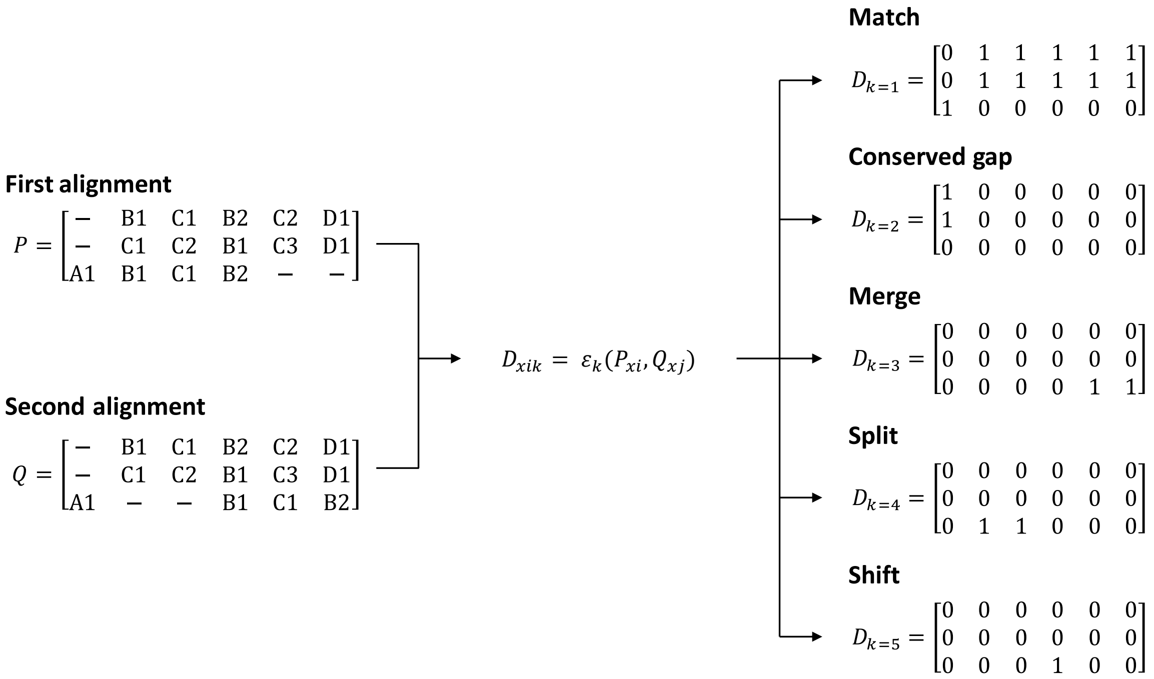

Figure S6 | Generating a dissimilarity matrix

For each column of *P*, the columns of Q with the highest S_{ij} is compared using the dissimilarity function defined in **Equation 3** to generate the dissimilarity matrix (*D*), which describes the dissimilarity of *Q* with respect to *P*.

Match

$$D_{k=1} = \begin{bmatrix} 0 & 1 & 1 & 1 & 1 & 1 \\ 0 & 1 & 1 & 1 & 1 & 1 \\ 1 & 0 & 0 & 0 & 0 & 0 \end{bmatrix}$$

Conserved gap

$$D_{k=2} = \begin{bmatrix} 1 & 0 & 0 & 0 & 0 & 0 \\ 1 & 0 & 0 & 0 & 0 & 0 \\ 0 & 0 & 0 & 0 & 0 & 0 \end{bmatrix}$$

Merge

$$D_{k=3} = \begin{bmatrix} 0 & 0 & 0 & 0 & 0 & 0 \\ 0 & 0 & 0 & 0 & 0 & 0 \\ 0 & 0 & 0 & 0 & 1 & 1 \end{bmatrix}$$

Split

$$D_{k=4} = \begin{bmatrix} 0 & 0 & 0 & 0 & 0 & 0 \\ 0 & 0 & 0 & 0 & 0 & 0 \\ 0 & 1 & 1 & 0 & 0 & 0 \end{bmatrix}$$

Shift

$$D_{k=5} = \begin{bmatrix} 0 & 0 & 0 & 0 & 0 & 0 \\ 0 & 0 & 0 & 0 & 0 & 0 \\ 0 & 0 & 0 & 1 & 0 & 0 \end{bmatrix}$$

$$R_{ki} = \frac{1}{n}\sum_{x=1}^{n} D_{xik}$$

Results

$$R = \begin{bmatrix} 0.33 & 0.67 & 0.67 & 0.67 & 0.67 & 0.67 \\ 0.67 & 0 & 0 & 0 & 0 & 0 \\ 0 & 0 & 0 & 0 & 0.33 & 0.33 \\ 0 & 0.33 & 0.33 & 0 & 0 & 0 \\ 0 & 0 & 0 & 0.33 & 0 & 0 \end{bmatrix}$$

Figure S7 | Generating a results matrix

The columns of the dissimilarity matrix (D) are summarised using the summary function described in **Equation 5** to generate a results matrix (R). Each row of *R* describes the column averages of a different *k* of the dissimilarity matrix. The columns when *k=5* are simply averaged (describing conserved gaps). All other columns are averaged and divided by the inverse of the column average of *k=5* (describing average dissimilarities as a proportion of the column that is not conserved gaps).

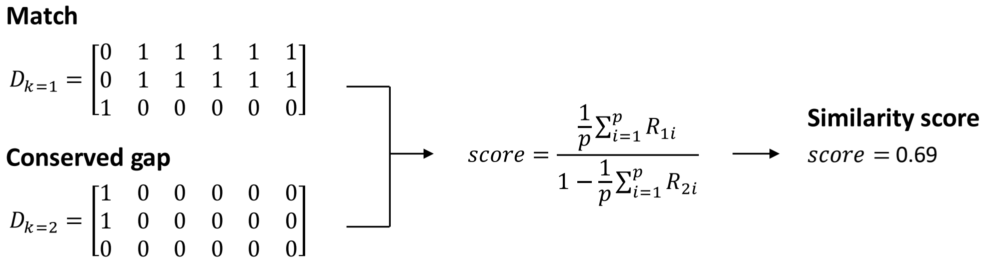

Figure S8 | Generating an overall similarity score

The dissimilarity matrix (D) is summarised using the summary function described in **Equation 6** to generate a final weighted average score, which measures the overall similarity of the initial MSAs. The score is the weighted average of matching characters out of the total characters that are not conserved gaps.
